# Migration of aortobifemoral bypass graft into the duodenum with extensive duodenal wall erosion: a rare case report

**DOI:** 10.1016/j.radcr.2025.09.068

**Published:** 2025-10-15

**Authors:** Halil İbrahim Altunbulak, Bilal Altunbulak

**Affiliations:** a Department of Radiology, Hacettepe University, Ankara, Altındağ Türkiye; b Department of Radiology, Bilkent City Hospital of Ankara, Ankara, Yenimahalle, Türkiye; c Department of Radiology, State Hospital of Tatvan, Bitlis, Türkiye

**Keywords:** Aortoenteric fistula, Erosion, Migration, Graft, Life-threatening

## Abstract

Secondary aortoenteric fistula (SAEF) is a rare but life-threatening complication following aortic graft placement, often leading to gastrointestinal hemorrhage or sepsis. We report a rare case of an eroded aortobifemoral bypass graft that migrated into the duodenal lumen, emphasizing its clinical presentation, radiological findings, and management approach.

## Introduction

Aortoiliac occlusive disease (AIOD) is a prevalent vascular pathology that manifests as intermittent claudication, erectile dysfunction, and, in severe cases, end-organ ischemia [1]. Surgical intervention with aortobifemoral bypass grafting is a well-established treatment modality; however, graft-related complications, such as infection, erosion, and secondary aortoenteric fistula, may occur postoperatively [2]. Aortoenteric fistula (AEF) is a rare but fatal complication characterized by an abnormal communication between the aortic graft and the gastrointestinal tract, most commonly involving the duodenum [3]. The incidence of SAEF has been reported between 0.36% and 1.6% in the literature [4]. We present a unique case of an eroded aortobifemoral bypass graft that migrated into the duodenal lumen because paraprosthetic graft erosion is considered a precursor lesion that may progress to overt fistula formation highlighting its clinical course, imaging features, and surgical management.

## Case presentation

Written informed consent was obtained from the patient in accordance with ethical guidelines.

**Fig. 1:**
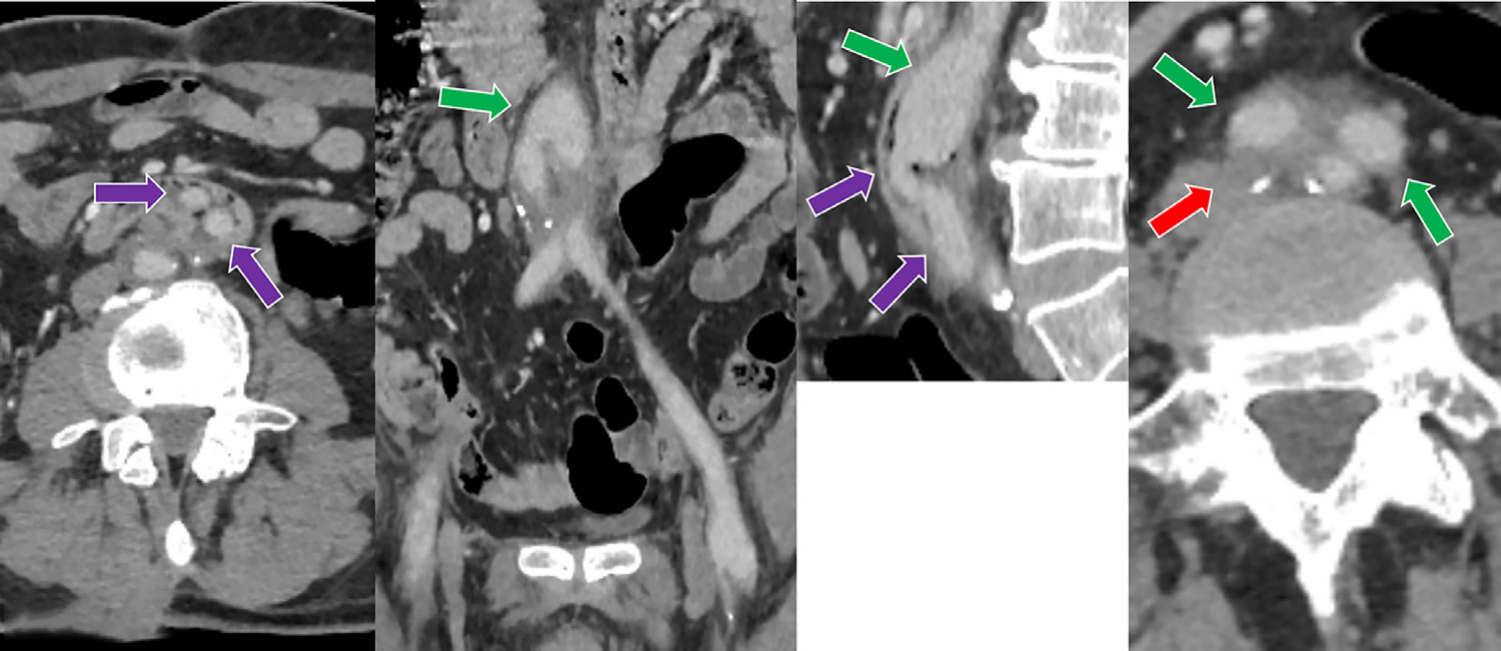
Computed tomography angiography (CTA) of a 47-year-old male demonstrating right common iliac artery occlusion (red arrow) and left external iliac artery occlusion (yellow arrow), both treated with a bilateral aortobifemoral bypass graft (green arrow). The image also shows migration of the graft into the third part of duodenal lumen (purple arrow), erosion of the right graft limb (blue arrow), and patency of the bilateral distal graft anastomoses (orange arrow). Perigraft increased density observed on the CT images, correlating with the patient’s clinical presentation of pain and elevated acute-phase reactants, is suggestive of a localized graft infection.

**Fig. 2:**
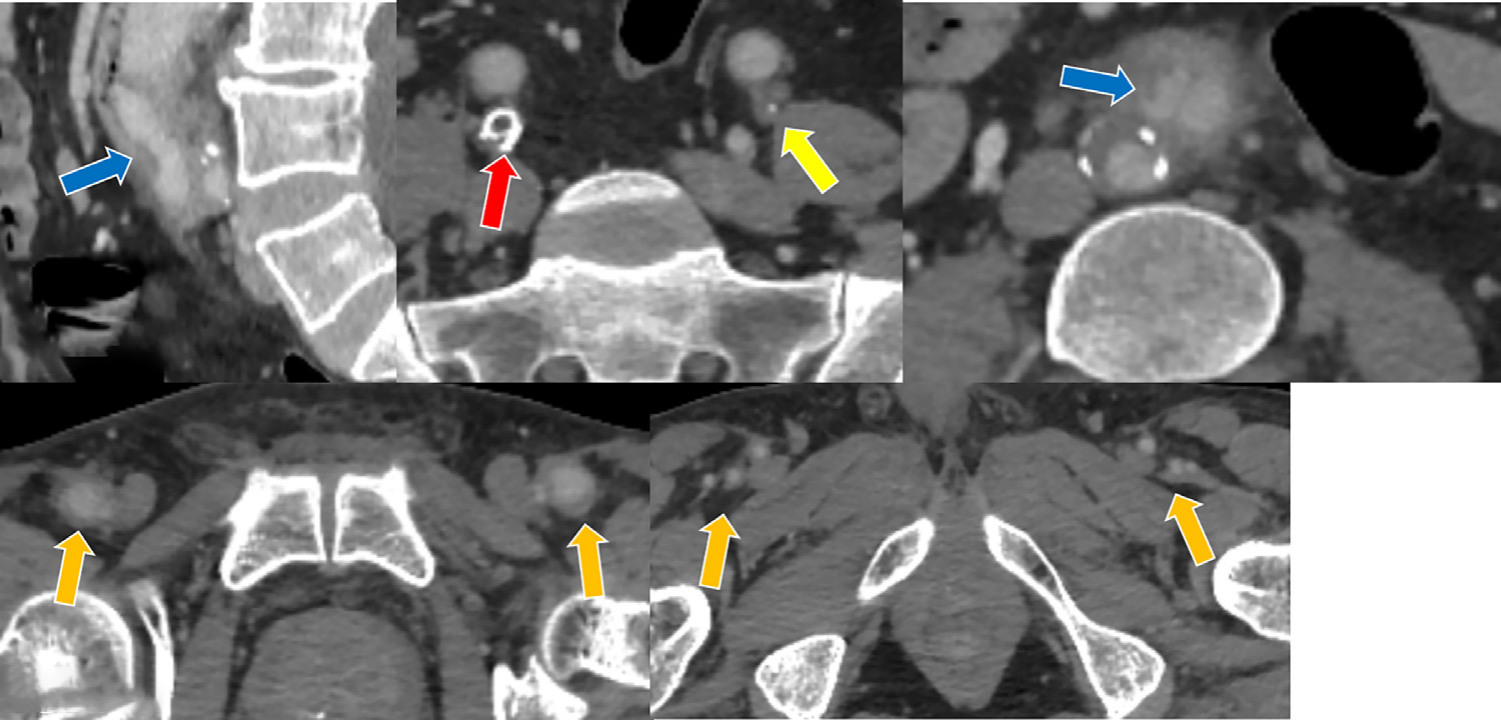
Computed tomography angiography (CTA) of a 47-year-old male demonstrating right common iliac artery occlusion (red arrow) and left external iliac artery occlusion (yellow arrow), both treated with a bilateral aortobifemoral bypass graft (green arrow). The image also shows migration of the graft into the duodenal lumen (purple arrow), erosion of the right graft limb (blue arrow), and patency of the bilateral distal graft anastomoses (orange arrow).

A 47-year-old male with a history of aortoiliac occlusive disease (AIOD), previous aortobifemoral bypass grafting, and chronic obstructive pulmonary disease (COPD) presented withabdominal pain . Laboratory findings revealed elevated acute-phase reactants. Computed tomography angiography (CTA) demonstrated paraprosthetic eroded duodenum withgraft segment protruding into the duodenal lumen (Fig. 1, Fig. 2, Videos 1 and 2). Subsequently, an endoscopic examination was performed, revealing a graft in the third part of the duodenum. The findings were consistent with those observed on CT (not shown).The patient was informed about the urgency of the condition and the need for immediate surgical intervention. However, despite medical recommendations, the patient declined surgical treatment.

## Discussion

Most reported cases of aortoenteric fistulas occur in male patients, reflecting the higher prevalence of abdominal aortic aneurysms and aortic surgery in men [5]. AEF is classified as either primary (arising from native aortic pathology, such as aneurysm) or secondary (resulting from previous aortic graft placement) [6]. SAEF is more commonly associated with infection or chronic mechanical erosion of the prosthetic graft against the adjacent bowel wall [7]. Paraprosthetic erosion, a precursor to SAEF, represents a stage where the graft erodes into the bowel wall without forming a direct fistulous connection [8].

The typical clinical manifestation of SAEF is gastrointestinal bleeding, ranging from minor “herald bleeds” to massive hemorrhage but nonspecific symptoms such as fever, unexplained abdominal pain, and sepsis may also occur [2]. In contrast, our patient presented only with progressive abdominal pain and elevated inflammatory markers, without gastrointestinal bleeding, underscoring the atypical and insidious nature of this case. Diagnostic imaging, particularly CTA, plays a crucial role in detecting signs of SAEF, including loss of fat planes between the graft and bowel, pseudoaneurysm formation, and contrast extravasation into the gastrointestinal tract [9]. In our case, CTA clearly demonstrated graft migration and duodenal wall erosion, later confirmed by direct endoscopic visualization.

The primary goal of treatment is hemorrhage control and infection management. The traditional surgical approach involves graft removal and extra-anatomic bypass, while alternative strategies include in situ graft replacement or endovascular aortic repair (EVAR) as a bridge to definitive surgery [10]. Regardless of presentation as either SAEF or paraprosthetic erosion, both conditions carry a high mortality rate and require prompt surgical intervention [11]. In our case, where paraprosthetic graft erosion is incidentally detected, an aggressive surgical approach is warranted to prevent progression to life-threatening AEF, however the patient declined operative treatment despite the life-threatening prognosis, emphasizing the importance of timely surgical decision-making in preventing progression to catastrophic outcomes.

## Conclusion

AEF remains a rare but critical complication following aortobifemoral bypass grafting. Clinicians should maintain a high index of suspicion in patients presenting with unexplained gastrointestinal bleeding or sepsis postoperatively. Given the similar management approach for SAEF and paraprosthetic graft erosion, early diagnosis using CTA and proactive surgical intervention are essential. In cases of incidental detection of paraprosthetic graft erosion, aggressive management is crucial to prevent the development of life-threatening AEF. This case highlights an atypical presentation, where the patient exhibited only progressive abdominal pain without overt bleeding and declined surgical intervention, emphasizing that early detection using CTA and proactive management are crucial to prevent progression to life-threatening AEF. Aggressive surveillance and timely intervention remain paramount even in incidentally detected paraprosthetic graft erosions.

## Patient consent

Written informed consent was obtained from the patient for publication of this case report and any accompanying images.

If applicable, identifying information has been omitted or anonymized to ensure patient confidentiality.

## Central Message

Aortoenteric fistula (AEF) and its precursor, paraprosthetic graft erosion, are rare but lethal complications following aortobifemoral bypass grafting, both carrying high mortality rates. Early diagnosis via computed tomography angiography (CTA) and aggressive surgical intervention are paramount for both conditions to prevent life-threatening outcomes and improve patient prognosis. Proactive and swift management is particularly crucial in cases of incidentally detected paraprosthetic graft erosion to avert progression to overt AEF.

## Appendix Supplementary materials

Image, video 1

Videos 1–2: Erosion of the third part of the duodenum and the right prosthetic limb, with migration of the aortobifemoral bypass graft into the duodenal lumen. Image, video 2
